# Shape control of size-selected naked platinum nanocrystals

**DOI:** 10.1038/s41467-021-23305-7

**Published:** 2021-05-21

**Authors:** Yu Xia, Diana Nelli, Riccardo Ferrando, Jun Yuan, Z. Y. Li

**Affiliations:** 1 School of Physics and Astronomy, University of Birmingham, Edgbaston, Birmingham, UK; 2 Department of Materials Science and Engineering, Southern University of Science and Technology, Shenzhen, Guangdong China; 3 Dipartimento di Fisica and CNR/IMEM, Università degli Studi di Genova, Genova, Italy; 4 Department of Physics, University of York, Heslington, York, UK

**Keywords:** Nanoparticles, Synthesis and processing

## Abstract

Controlled growth of far-from-equilibrium-shaped nanoparticles with size selection is essential for the exploration of their unique physical and chemical properties. Shape control by wet-chemistry preparation methods produces surfactant-covered surfaces with limited understanding due to the complexity of the processes involved. Here, we report the controlled production and transformation of octahedra to tetrahedra of size-selected platinum nanocrystals with clean surfaces in an inert gas environment. Molecular dynamics simulations of the growth reveal the key symmetry-breaking atomic mechanism for this autocatalytic shape transformation, confirming the experimental conditions required. In-situ heating experiments demonstrate the relative stability of both octahedral and tetrahedral Pt nanocrystals at least up to 700 °C and that the extended surface diffusion at higher temperature transforms the nanocrystals into equilibrium shape.

## Abstract (web summary)

Tetrahedral nanocrystals are out-of-equilibrium structures whose growth mechanism is a long-standing open problem. Here, the authors show that pure Pt tetrahedral nanocrystals grow in the gas phase and single out the defect-mediated mechanism leading to the symmetry-breaking for tetrahedral growth.

## Introduction

The properties of nanoparticles are known to strongly depend on both size and shape^1,2^. Therefore, producing nanoparticles with controlled size and shape is extremely important for applications. With this respect, the mechanistic understanding of the shape control is essential for turning shape-directed nanoparticle synthesis from an art to science.

Depending on the degree of non-equilibrium of the growth, shape-directed nanocrystal synthesis was rationalized so far on the basis of either the Equilibrium Wulff Construction (EWC) or the Kinetic Wulff Construction (KWC)^3–5^. In EWC, nanocrystal shape is determined from the equilibrium surface free energies of facets with different orientations. In KWC, nanocrystal shape derives from the different growth velocities of these different facets. Clearly, the competition between octahedral and tetrahedral growth can neither be explained by the EWC nor by the KWC. Both octahedra and tetrahedra are non-equilibrium shapes (see the calculations of Supplementary Fig. 1 and Supplementary Table 1), with quite unfavourable surface-to-volume ratios, so that their growth cannot be predicted by the EWC. On the other hand, both structures expose the same type of (111) facets only, so that the simplistic orientation-directed growth model of the KWC does not apply. In fact, in order to form a tetrahedron, only four symmetrically placed (111) orientations over the possible equivalent eight (111) orientations must grow. In an isotropic environment, with atoms landing on the nanocrystals randomly from all directions, there is no a priori reason to select this reduced set of growing orientations. The explanation of this long-standing open problem^6–9^ thus hints at a subtler growth mechanism that we are going to present here.

A first important step in understanding this issue was made by Wang et al.^8^, who showed that the growth of tetrahedra takes place at a stronger non-equilibrium preparation condition than that of octahedra. They noted also that a larger tetrahedron can grow from a smaller octahedron. However, they did not unravel the atomic-level mechanisms causing this type of growth, which, as we will show below, requires a peculiar type of symmetry breaking. In the experiments of Wang et al.^8^, the situation was indeed difficult to analyse at the atomic level due to the complexity of the processes occurring in their wet-chemistry synthesis.

Pt is an excellent catalyst for many industrially important chemical reactions. Although far-from equilibrium tetrahedra have not been extensively studied due to their rarity, there are already experimental evidences that they are particularly useful for certain catalytic reactions^2,6^. Recent advancements in preparation of realistic quantities of catalytic nanoparticles with size-selection by gas phase deposition^10,11^ have opened a way for us to study the shape-induced effects of nanocatalysts in a systematic way. Tetrahedral Pt nanoparticles would also exhibit unique plasmon responses^12^ that can be explored in bio-sensing and spectroscopy of trace elements. The availability of size-tunable tetrahedral nanoparticles is therefore not only of fundamental interest, but of enormous practical values.

In this study, we tackle this problem by taking advantage of the clean and relatively simple environment of gas-phase growth using the magnetron-sputtering technique^13,14^. We report a controlled gas-phase growth of octahedral and tetrahedral Pt nanocrystals with mass selection in terms of number of atoms up to 20,000 (with corresponding diameter around 9 nm). Even though tetrahedra are the most out-of-equilibrium among the platonic shaped nanocrystals, we show that they can be optimized to be a dominant product, as confirmed by our atomic scale Scanning Transmission Electron Microscopy in high-angle annular dark field mode (HAADF-STEM). This is achieved without the use of precursors or surfactants. We therefore show that the growth of Pt tetrahedra and octahedra in the gas phase can arise from driving forces inherent to the kinetics of the pure metal condensation which can be controlled experimentally. This is supported by our atomic-scale simulations, which not only reproduce the main features of the experiment, but also unravel the underlying atomic-scale mechanism. The analysis shows the key role of atomic mobility between facets and of the generation of specific metastable defects during growth and gives support to the optimal experimental parameters. It is also confirmed by an in situ heating experiment where surface diffusion can be manipulated.

## Results

### Growth experiments: octahedral vs tetrahedral structures

**Fig. 1:**
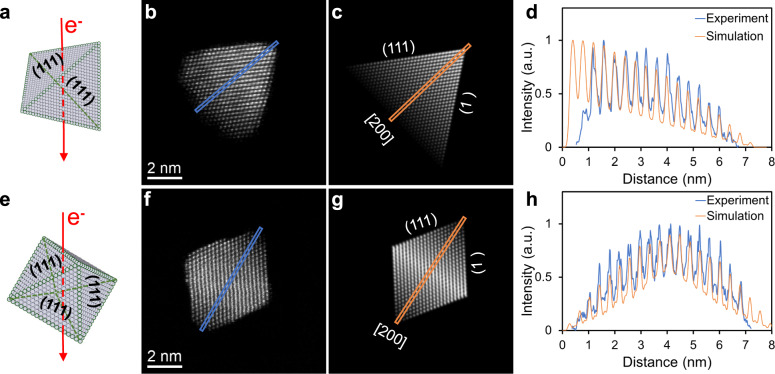
The typical shaped Pt_5000_ nanoparticles imaged using HAADF-STEM. **a**–**d** Tetrahedral nanocrystals, **e**–**h** octahedral nanocrystals. **a**, **e** Schematic representation of tetrahedral and octahedral nanocrystals with their characteristic (111) facets. The red lines indicate the direction of the electron (e^-^) beam travelling along the [011] zone axis of the nanocrystals. The experimental images (**b**, **f**) of these nanoparticles are compared with the corresponding simulation (**c**, **g**) based on the idealized models of tetrahedron and octahedron as shown in the first column, respectively. The (111) facets are indicated as well as the [200] direction along which the intensity line profiles are compared in (**d**) and (**h**), respectively. Both the image and line profile comparisons show an overall agreement, except that the real nanocrystals have truncated vertices.

Figure 1 shows atomic scale HAADF-STEM imaging of two most abundant shaped nanoparticles, namely tetrahedron (Fig. 1, a–d) and octahedron (Fig. 1, e-h), with a nominal mass of 5000 Pt atoms, found in a cluster beam generated by a magnetron sputtering inert gas aggregation source. The details of the source are described later as well as in the Methods section. The experimental images in Fig. 1(b, f) are taken with the electron beam travelling down an [011] zone axis of the tetrahedral and octahedral nanoparticles respectively, as shown in Fig. 1(a, e) respectively. Two simulated STEM images are displayed in Fig. 1c and 1g, where the atomic column intensities can be used as a guide to the local thickness variation^15,16^. For shape identification, our experience has shown that there is no need to take into consideration small structural relaxation that may be present^17^. Close matches of both the outlines of the particles and the overall atomic column intensity variations between the experimental and simulated images demonstrate that the nanoparticles have the expected shapes. However, for example in Fig. 1b, the top-right corner of the experimental image for tetrahedron-shaped nanoparticle shows that the atoms are missing from the edge when viewed edge-on, and the lower-left image of the topologically equivalent edge shows that the missing atoms decorate the edge unevenly lengthwise, particularly at vertices. These phenomena indicate that atoms at edges and vertices are highly unstable, but they do not lead to smooth regular truncated facets. The result is also confirmed quantitatively by line profiles drawn across the images of nanoparticles concerned, as shown in Fig. 1(d, h) respectively. The experimental and simulation line profiles mostly match each other, apart from those parts at the beginning and at the end, due to the ‘missing atoms’ at the edges and vertices. This is consistent with the total energy estimate for tetrahedral-shaped nanoparticles with different degrees of vertex truncations (see Supplementary Fig. 1 and Supplementary Table 1 for the excess energy of differently shaped nanoparticles), with the total energy for an ideal tetrahedron being much higher than those for the octahedral-shaped particles which can be taken to be vertex-truncated tetrahedron where the four {111} surfaces created by vertex-truncation equal to that of the original {111} facets.

**Fig. 2:**
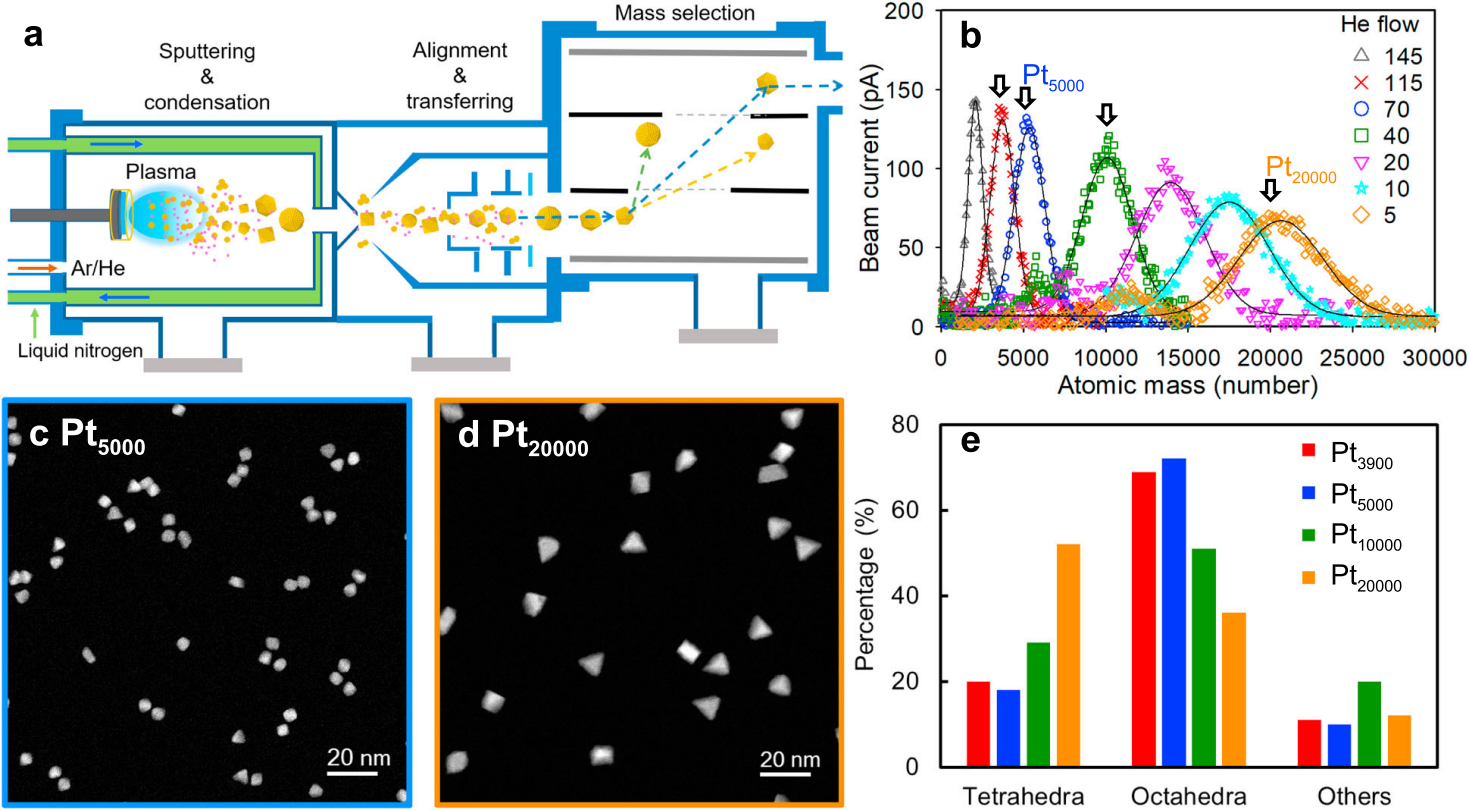
Controlling size and shape of Pt nanoparticles. **a** The schematic drawing of magnetron sputtering inert gas condensation beam source used for producing Pt nanoparticles. **b** Mass spectra of Pt nanoparticles produced with the Ar flow rate fixed at 90 sccm and varied He flow rates from 5 to 145 sccm. HAADF-STEM images of (**c**) Pt_5000_ nanoparticles when He flow is at 70 sccm, and (**d**) Pt_20000_ when He flow is at 5 sccm, respectively. The corresponding percentages of Pt nanoparticles with certain shapes were shown in (**e**), together with the data from Pt_3900_ and Pt_10000_ samples (with corresponding He flow at 115 and 40 sccm, respectively). Data were taken, with the mass selector, at the peak position of the size distribution as shown in (**b**). Other parameters used: sputtering power of 135 W and the condensation length of 250 mm.

As the truncated-octahedral nanocrystals are the most stable at thermal equilibrium (see Supplementary Discussion SD-1, Excess energy of different structural motifs), the growth of octahedral and tetrahedral nanocrystals must be due to kinetic processes which open ways to experimental manipulations of the different shapes. Figure 2a shows a schematic of the cluster source used in this study. Here, the nanoparticles are generated by magnetron plasma sputtering of a Pt target in an Ar/He mixture environment and are grown as they travel towards the exit of the condensation chamber. They are then extracted through a nozzle and positive charged particles are focused and accelerated through the optics and mass selected by a lateral time-of-flight (ToF) mass spectrometer. In this set-up, we can select the size of nanoparticles easily. For example, for Pt_5000_ and Pt_20000_ nanoparticles, we have 5000 ± 150 and 20,000 ± 600, respectively, when the mass (*M*) resolution of ToF was set at *M*/Δ*M* of 16. However, the shape of the nanoparticles will depend on many factors which determine how the particles are formed. The operation parameters that can be varied here are power of the sputtering, argon (Ar) and helium (He) gas composition and their flow rates while keeping the condensation length the same.

Figure 2b shows the experimental result of a simple kinetic study by mass spectroscopy in which the only kinetic variable is the He flow rate in the Ar/He inert gas mixture introduced to the condensation chamber. In this case, the sputtering power is kept at a relatively large value of 135 W and the Ar flow rate is kept constant at 90 standard cubic centimetres per minute (sccm). Here, the main result of increasing the He flow rate is the decrease of the mean value of the nanoparticle size as well as the number of particles detected (as represented by the area under the main peak of the mass spectrum). To the first approximation, the inverse of the He flow-rate can be interpreted as proportional to the dwell time of the nanoparticles while traversing the condensation chamber. In Supplementary Discussion SD-2 (Influence of He flow rate on the formation of Pt nanoparticles), we show that, initially, the number of the nanoparticles detected grows quickly with the transit time, then stabilizes at roughly a constant value, which indicates that the He flow rate we have applied is sufficiently small that has little effect on the number of nanoparticles in the beam. Interestingly, the mean particle size increases throughout the full range of the nanoparticle transit times and the evolution can be fitted to a single exponential relationship. This phenomenon suggests strongly that the increase in particle size is likely due to adatom condensation on the existing nanoparticle nuclei which is enhanced due to longer effective transit time^18^. We also see that the mass spectra are largely symmetrically distributed, closer to Gaussian than log-normal distribution, suggesting that the possibility of nanoparticle size increase through He-enhanced two-body collision of Pt nanoparticles is also small^19^. Particle-particle aggregation would often lead to either elongated or fractal shaped clusters^20^, which are not observed here.

We thus can study the growth pathway of the shaped nanoparticles in the adatom adsorption region by examining the shape distribution of the size-selected particles at different He flow-rates. This is done for the nanoparticle size at 3900, 5000, 10,000 and 20,000 when the He flow-rate is at 115 sccm, 70 sccm, 40 sccm and 5 sccm, respectively (see examples in Fig. 2c, d and Supplementary Discussion SD-3, Procedures of capturing images for shape identification and their uncertainty). The shape distributions at 115/70 sccm would be more representative of the particles soon after nucleation near the plasma zone as they are quickly swept through the condensation chamber so opportunity for further growth is limited. The shape distribution at 5 sccm would reflect the further growth of these nuclei as they travel through the condensation chamber at a more leisurely pace. The details of how images are captured for shape identification and their uncertainty are described in Supplementary Discussion (SD-3, Procedures of capturing images for shape identification and their uncertainty). The shapes of the particles are inferred by comparing the experimental HAADF-STEM images of the nanoparticles with that in a library (see Supplementary Discussion SD-4, Tetrahedron and Octahedron Image Libraries) of simulated HAADF-STEM images of ideal tetrahedral and octahedral fcc nanocrystals of comparable sizes at all possible orientations, as we have demonstrated in Fig. 1.

The most prominent finding, shown in Fig. 2e, is that the dominant shape of the Pt_5000_ nanoparticles grown is octahedral (73% of 616 nanoparticles counted) while the dominant shape of the Pt_20000_ nanoparticles is tetrahedral (52% of 753 nanoparticles counted) (for details, see Supplementary Table 2). The results for Pt_10000_ nanoparicles are in between. It seems that as the size increases, there is a higher percentage of particles with the tetrahedron shape. In other words, at least some of the tetrahedral Pt are grown from the smaller octahedral Pt. However, this growth cannot be easily understood by the classical KWC mechanism, which depends on the varied growth rates of different facets^21,22^. Both tetrahedral and octahedral nanoparticles are surrounded by identical (111) facets. Furthermore, tetrahedral nanoparticles are more out-of-equilibrium than the octahedral nanoparticles (see Supplementary Discussion, SD-1, Excess Energy of Different Structural Motifs), therefore the observed shape transformation also cannot be driven by energetics. A similar conundrum exists for the growth of tetrahedral nanoparticles in solutions and has remained unresolved^22^. Before we discuss the resolution of this puzzle in our gas phase growth below, we should also point out that those nanoparticles classified as “others” in Fig. 2 consist of some single or multiply twinned Pt particles or hexagonal shaped particles or particles with a long axis (see Supplementary Fig. 6). The absolute numbers of “other” nanoparticles are small and comparable in all measured samples, so they may not play significant roles in the growth pathway from octahedra to tetrahedra. Anyway, this does not exclude the possibility that a few tetrahedra may grow without passing through the octahedral structure, but our data indicate that the octahedral route is dominant.

### Growth simulations: defect-mediated symmetry breaking

**Fig. 3:**
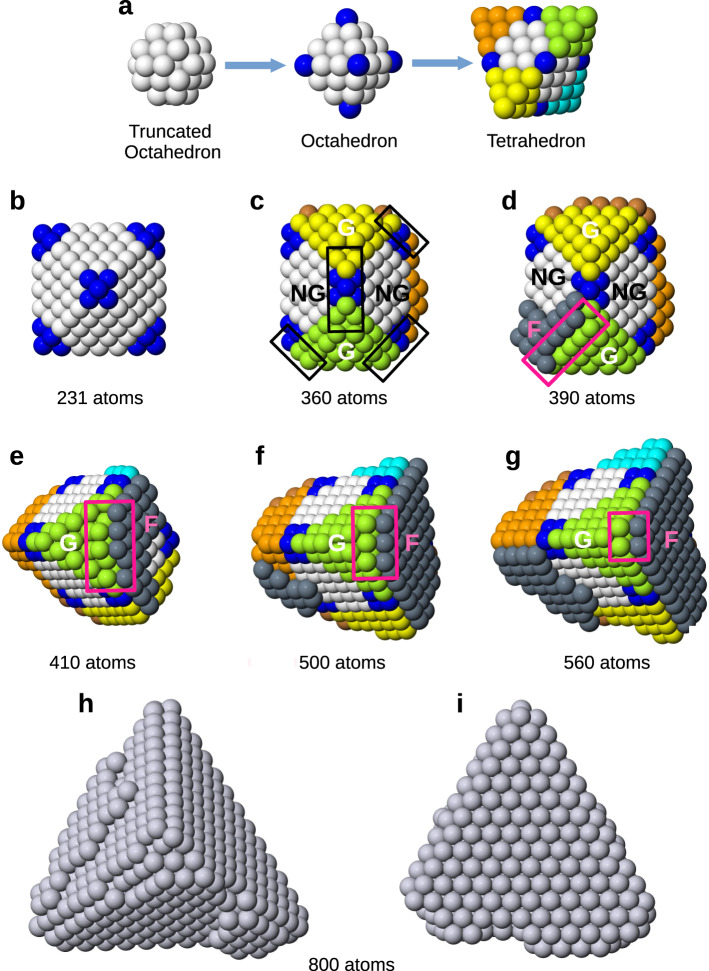
Growth pathway from truncated octahedral to octahedral and to tetrahedral shaped nanoparticles. **a** Schematic representation of the two main steps of the growth sequence. A truncated octahedron (white atoms) becomes an octahedron after growth on all (100) facets to complete six vertices (blue atoms). The tetrahedron is then obtained by growing equivalent tips on only four (111) facets of the octahedron over eight, in such a way that octahedral symmetry is broken with a specific pattern. These tips are colored in y ellow, green, cyan and orange. **b**–**g** Snapshots taken for a growth simulation at 400 K and deposition rate of 0.1 atom/ns. (**b**-**d**) and (**e**-**g**) show the same growing nanoparticle from different perspectives. The simulation is started from a truncated octahedron of 201 atoms (white atoms). **b** Octahedral vertices are completed. **c** Non-neighbouring growing (G, in yellow and green) facets are formed, separated by non-growing facets (NG). Small tetrahedral edges are formed (enclosed in black rectangles). **d** An island in stacking fault (F, in dark grey) starts to grow at the corner between G and an NG facets, creating a (100)-like fourfold sites (enclosed in the pink rectangle) on which (**e**) new atoms adsorb and facilitate the growth of the tetrahedral tips by a self-replicating process (**f**) which finally leads to the tetrahedral nanoparticle (**g**), which continues to grow as a tetrahedron (**h**, **i**).

We take advantage of the relatively clean environment of the gas phase growth to study the atomic processes behind the octahedral-to-tetrahedral shape transformation using molecular dynamics. Our results show that there is a kinetic growth pathway in which the nanoparticles progressively move further and further away from equilibrium, while growing in the context of high-symmetry structural motifs. This pathway comprises two steps: (a) truncated octahedron → octahedron; (b) octahedron → tetrahedron. These steps are schematically shown in Fig. 3a. Small nuclei formed at the high temperature atomic vapour at the sputtering target are mostly truncated octahedra because of fast equilibration^23,24^. They can become octahedra after growth on all six (100) facets to complete their vertices, through adatom adsorption^25^. Then, the further transformation into tetrahedron can only be obtained by growing tips on four (111) facets of the octahedron out of eight, in such a way that octahedral symmetry is broken in a very specific pattern. Detailed analysis of the atomistic steps involved shows how that is achieved.

We believe that the initial nucleation of nanoparticles in the presence of energetic plasma will result in nearly equilibrium shapes which are truncated octahedra^23,26^, which present both (111) and (100) facets. Adsorption on (100) facets is more energetically favourable than on (111) facets, by about 0.5 eV in our model. It is well known that atoms deposited on (111) facets can move to the (100) facets where they get trapped, thus growing the octahedral tips^25^. This growth step follows the classical KWC model, with the out-of-equilibrium growth driven by the growth rates of different facets: (100) facets grow faster so that they tend to disappear. This explanation is consistent with the dominance of octahedra in the Pt_3900_ and Pt_5000_ samples we found experimentally when the flow rate was high such that Pt nanoparticles newly nucleated at the plasma sputtering target were quickly swept through the condensation chamber (see Fig. 2e).

Once an octahedron is formed, how can its O_h_ symmetry be broken and a tetrahedron (T_d_ symmetry, a subgroup of O_h_) grow steadily on top of it? As all facets are equivalent in both shaped nanocrystals, of (111) type, we therefore cannot in principle assume different growth rates. Some more subtle mechanisms should come into play in this further growth step, which we explain with the aid of representative snapshots from a molecular dynamics simulation of growth of a Pt_231_ octahedral nanocrystal (Fig. 3(b–g), see the Methods section for the simulation method used). We first analyse the growth simulations of such small nanocrystals because they allow us to describe more easily the growth mechanisms by following the process atom by atom. Simulations of the growth of larger nanocrystals, of sizes in the range of the experiments, are discussed later here and in Supplementary Discussion SD-6 (Molecular Dynamics Simulations for Different Sizes and Temperatures).

The simulation originally starts from a truncated-octahedral structure, which becomes the octahedron by completing its six vertices (Fig. 3b). A crucial ingredient for the subsequent tetrahedral growth is the mobility of atoms between nearby facets: in our simulations we find that it occurs by exchange and is activated already at relatively low temperatures. When the growth starts on a given facet of the octahedron, steps are created on that facet. These steps work as traps for diffusing adatoms. Since mobility from nearby facets is activated, atoms deposited there are likely to diffuse to the growing facet to stick there. We believe that this depletion effect renders the nucleation of islands on nearby facets less likely and leads to configurations of the type shown in Fig. 3c, in which initially growing (G) facets are separated by initially non-growing (NG) facets, which indeed may grow but at a lower rate than G facets, for the reason given below.

The growth of a layer on a G facet initiates the formation of a tetrahedral edge in its nearby NG facets, as shown in Fig. 3c. These sharp tetrahedral edges present favourable sites for adatom adsorption on the NG facet. In general, adsorption of atoms on facets is more favourable in the vicinity of edges, as known from experimental and computational results on Pt(111) surfaces^27,28^. Some small islands are therefore likely to nucleate on NG facets, with preferential placement at corners with G facets, where they can keep a rather compact shape while placing several atoms on edge sites (see Fig. 3d). An experimental example of such a compact island forming over an edge in octahedral nanocrystals can be seen in the top-left side of the octahedral nanocrystals shown in Fig. 1f. However, the formation of such an edge-bound step alone is not able to account for the shape transformation into a tetrahedron.

The key symmetry-breaking step starts if the island at the corner of the NG facet is in stacking fault, which creates fourfold adsorption sites on the G facet (Fig. 3d). These sites act as traps for new incoming adatoms (Fig. 3e), which contributes to locking the island in fault position. These adatoms create further new fourfold adsorption sites, causing an autocatalytic self-replicating process which can lead to the fast growth of a tetrahedral tip (Fig. 3f, g). By comparison, the island growth of the unfaulted layer will be slow and self-limiting (see Supplementary Figs. 7 and 9). The fast kinetics of tip growth over the layer-by-layer growth drives the shape transformation from an octahedron to a tetrahedron, because the symmetric placement of the corners between G and NG facets naturally leads to the growth of four non-neighbour facets over eight, finally producing the tetrahedral shape of Fig. 3h, i.

Growth sequences from further simulations at temperatures of 600 and 800 K, as well as the final results of our 70 independent simulations for the same sizes, are reported in the Supplementary Table 3 and Supplementary Figs. 7–9. We note that the metastable islands in fault position can revert back to fcc stacking during the growth process (see Supplementary Figs. 7–8). When this happens, the growth of nearby tetrahedral tips significantly slows down (see Supplementary Fig. 7), so that the tips remain truncated. The fact that faulted islands easily revert back to the natural stacking may also explain why we did not experimentally observe them in the final tetrahedral nanocrystals in our electron microscopy observations. On the other hand, stacking fault islands are indeed visible in Pd tetrahedra grown in the liquid phase (see the image in Fig. 3a of ref. ^8^), where those islands may have been stabilized by ligands after growth completion. This also indicates that the proposed growth mechanism is possible in liquid-phase synthesis. Further simulations were done to reach larger growth sizes, up to 3000 atoms (Supplementary Fig. 10) and to more than 14,000 atoms (Supplementary Fig. 11), which is in the same size range as those studied in experiments. These large-size simulations confirm that the transitions from truncated octahedra to octahedra and then to tetrahedra take place by the same growth mechanisms shown in Fig. 3. We note that the simulations of Supplementary Fig. 11 were run at a slightly higher temperature to allow octahedral growth up to large sizes within the limited simulation time scale.

The overall picture arising from our simulations is that nanocrystals initially grow close to their equilibrium shape, until they reach a critical size at which they are not able to equilibrate anymore. In fact, the larger the nanoparticle size, the slower the equilibration of its shape. After that critical size, kinetic trapping begins to dominate, so that truncated octahedra grow into octahedra and then, for even larger sizes, the octahedra grow into tetrahedra. This critical size depends both on the growth time scale and on temperature, being small for short growth times and for low temperatures. It can be increased in two ways, namely by growing on longer time scales and by increasing the temperature.

### Annealing experiments and simulations

**Fig. 4:**
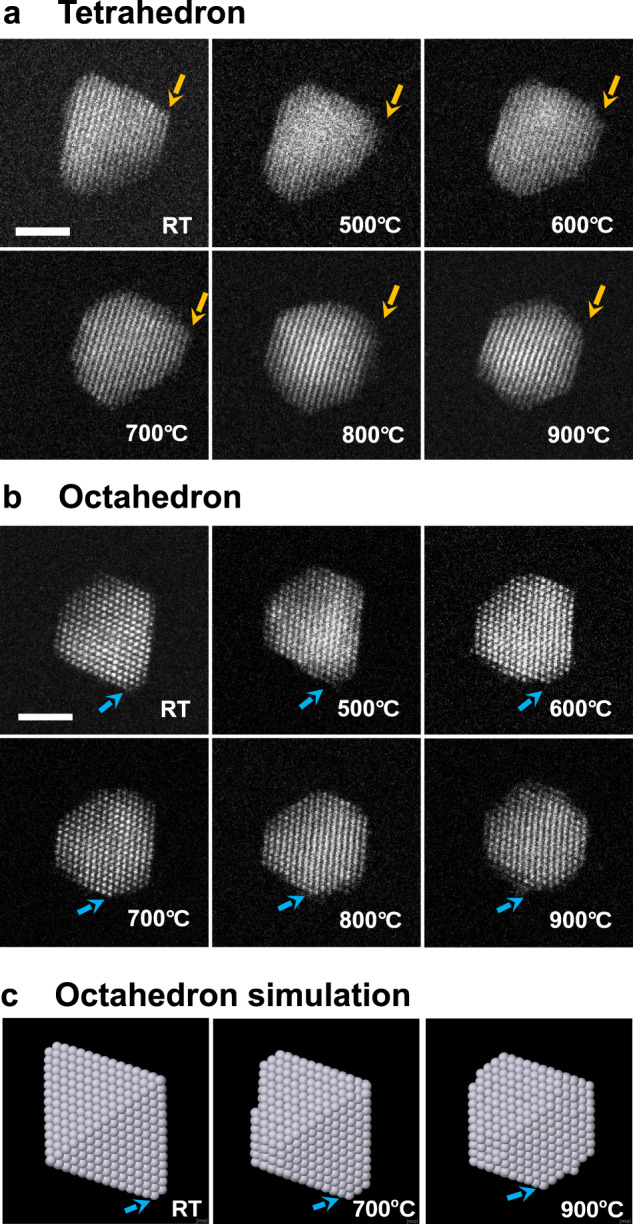
Direct visualisation of thermal transformation of Pt_2057_ tetrahedral nanoparticles with in situ heating experiment and comparison with the simulations. HAADF-STEM images of (**a**) tetrahedral Pt_2057_ and (**b**) octahedral Pt_2057_, quenched from various high annealing temperatures as indicated. **c** Simulation of the annealing of an octahedral Pt_2030_ structure, by heating up from room temperature (RT) to 700 and 900 °C and cooling back to RT, with rates of 1 K/ns (heating) and −1 K/ns (cooling). The simulation is indicative of the trend of structural evolution, which agrees with the experiment. The arrows indicate the truncated tips. In total, 2 nm-scale bars are used for all the experimental and simulation images.

An implicit condition for the proposed growth mode is that atomic mobility between nearby facets is activated, yet full mobility of atoms around the nanoparticle has not yet occurred. The latter would bring growth closer to equilibrium, resulting in a round or truncated octahedron. This has been verified both by annealing experiments and by annealing simulations, finding that truncated octahedral structures are indeed produced. Figure 4 displays HAADF-STEM images for Pt_2057_ nanoparticles after in situ heating from room temperature to 900 °C. The electron beam is incident along the [011]-zone axis as in Fig. 1. The samples were heated to the elevated temperature as indicated in the images and held there for 1 min, followed by quenching down to room temperature when the STEM images were acquired. It shows clearly that the tetrahedron is stable at least until 700 °C, even though some rearrangements of the atoms are observed. Interestingly, not only do we see the well-formed tetrahedral tip at the top of the image becoming rounded at 500 °C, but also the less-well-formed tetrahedral tip on the right of the image grows a little bit, indicating the surface diffusion activated by the modest heating promotes this autocatalytic growth. Further heating to 800 and 900 °C, a rounded shape of the nanoparticle can be seen following more substantial mass movement. The observation of the initiation of mass transport at various parts of the surface of the nanocrystals, occurring at different temperatures, suggests heterogeneity of surface diffusion barriers. This is the window of opportunity we explored experimentally to exercise shape control. At the same temperature of 900 °C, the octahedral Pt particle too is transformed to the rounded shape as shown in Fig. 4b. This result is nicely confirmed by annealing simulations (Fig. 4c) which are able to equilibrate the octahedron to a truncated octahedral shape at 900 °C even within the shorter time scale of simulations. The results again show that both tetrahedra and octahedra are not thermodynamic equilibrium states. They had been kinetically trapped in and they could thermally reach a stable structure above 700 °C on our annealing time scale. The high trapping temperature is characteristic of Pt^29,30^ which is good for the practical application of these shaped nanocrystals.

## Discussion

Compared with the complex and somewhat confused situation about the mechanisms for the growth of tetrahedra by wet chemistry^7,31,32^, the clean surface of nanoparticles generated in a vacuum system offers a simpler system for our simulation study of the atomic steps involved during tetrahedral growth. In particular, the nucleation of 2D islands at the edges of the nanoparticles, with hexagonal stacking on the underlying (111) facets seems to be the dominant processes involved because of its efficiency compared with random nucleation of fcc stacked islands in the middle of facets (see the growth rate simulation shown in Supplementary Discussion SD-7, Growth Simulation at 600 K and flux of 0.1 atoms/ns). The similar adatom growth mechanism should prevail for nanocrystals of other fcc noble metal systems. Our simulations show that this is indeed the case for palladium (Pd). This suggests that the controlled preparation of alloyed or core-shell bimetallic tetrahedral nanocrystals are possible using the clean gas-phase plasma synthesis method, further increasing the tunability of Pt-based nanoparticles^26,33^.

The key to the controlled gas-phase growth of tetrahedral fcc nanocrystals is a growth environment favouring predominance of adatom growth over particle-particle aggregation and finite diffusion length of adatom on the nanoparticle. Although the growth condition in a magnetron sputtering inert gas condensation chamber is a complex function of many factors, the understanding of the detailed atomistic mechanism gives us physical guidelines to their optimization. For example, particle-particle coalescence and secondary nucleation by inert gases can be suppressed by working with a low He vapour pressure as we did. The finite diffusion length can be controlled by transit time through the condensation chamber. The transit time can be adjusted via a combination of suitable condensation length, or as we have done in this work, by varying helium gas flow rates. The high yield gas phase synthesis route^11,14^ has the added advantages of producing clusters with bare surfaces, allowing the surface reactivities to be probed directly^10^ without the potentially shape-transforming steps of surfactant removal^34^ for nanoparticles produced by wet-chemistry approach. We expect that the demonstrated ability to manipulate operation parameters to select not only the size, but also the shape and the composition can play an important role to gain understanding of the physical mechanisms behind their often remarkable physical and chemical properties, paving the way for the rational design and controlled growth of bespoken nanoparticles for catalysts or optical devices.

## Methods

### Nanoparticle preparation

Pt nanoparticles are fabricated using DC magnetron sputtering inert gas condensation^14^. A mixture of argon and helium gases with individually controlled flow rate is introduced into the chamber for plasma sputtering of Pt targets (99.99%) as well as nanoparticle growth in the gas phase. The growth is terminated once they are out of the condensation chamber. The positively charged nanoparticles are focused and accelerated before their size selection through a lateral time-of-flight (ToF) mass filter. In the present study, the mass resolution (*M*/Δ*M*) is set to be 16. The Pt nanoparticle deposition is performed at a kinetic energy of 1200 eV.

### Characterisation and structural identification

Pt nanoparticles are imaged by a 200 kV JEM-2100F Transmission Electron Microscope (JEOL, Japan), which is equipped with a probe spherical aberration corrector for STEM (CEOS, Germany). The probe convergence angle is 19 mrad and the collection angle range of the high-angle annular dark field (HAADF) detector is set from 62 to 164 mrad. HAADF-STEM images are captured with an electron probe size of 8 C and a pixel dwell time of 38 μs with a 512 × 512 pixel scanning area. Atomic simulation environment (ASE)^35^ is used to generate the atomic models of Pt nanoparticles used in the kinetic simulation of the HAADF-STEM images of Pt nanoparticles^36^. For shape identification, the structural models used in Fig. 1 are idealized tetrahedron and octahedron.

### In situ heating experiments

Pt nanoparticles are deposited onto Wildfire nano twist chips covered with amorphous silicon nitride (SiN) support films (DENSsolutions, Netherlands), which can withstand heating up to 1300 °C. During the in situ experiment, the chips are heated to the required temperatures with a pre-set heating rate of 1000 °C/min. After holding at the annealing temperature for 1 min, the chips are quenched to room temperature with a cooling rate of 2000 °C/min. The Pt nanoparticles are examined by HAADF-STEM imaging, with a beam current density of 15 pA/cm^2^, a pixel dwell time of 10 μs and an image size of 512 × 512 pixels. The resultant electron dose rate is controlled at 0.7~1 × 10^4^ electrons/Å^2^ per frame.

### Simulation methods

Molecular Dynamics (MD) growth simulations are made by molecular dynamics using the same type of procedure adopted in ref. ^26,37^. Simulations start from a truncated octahedral structure of 201 atoms. Atoms are deposited one by one isotropically from random directions every 1 and 10 ns, corresponding to deposition rates of 1 and 0.1 atoms/ns. Simulations are stopped after the deposition of about 800 atoms, i.e., at nanoparticle size of about 1000 atoms. The equations of motion are solved by the Velocity Verlet algorithm with a time step of 5 fs. Temperature is kept constant by an Andersen thermostat. Seven different temperatures from 300 to 900 K are considered. For each temperature and deposition rate, 5 independent simulations are made. Annealing and quenching MD simulations are performed by heating up the nanoparticles in steps of 1 K every ns. Some more simulations for larger sizes (up to more than 14,000 atoms) were also done, at the deposition rate of 1 atom/ns and for temperatures between 700 and 900 K. Pt-Pt interactions are modelled by the Gupta potential^38^. Form and parameters of the potential are given in ref. ^30^, where it has been demonstrated that the potential is able to reproduce the experimental growth structures of PtPd alloy nanoparticles both in the Pt-rich and in the Pd-rich cases. In order to evaluate the energetic stability of the structural motifs, we have calculated the excess energy *E*_*exc*_ defined as^24^:

1 $${E}_{exc}=(E+N\varepsilon )/{N}^{2/3}$$

where *ε* is the cohesive energy per atom in bulk Pt, *E* and *N* are the binding energy and the number of atoms of the nanoparticle.

## Supplementary information

Supplementary Information

## Data Availability

All experimental and simulation data in the main text and the supplementary materials are available upon request to the authors.

## Code availability

Molecular Dynamics codes used in the simulation are available upon request to ferrando@fisica.unige.it.
