# Supplementary material for: Shape control of size-selected naked platinum nanocrystals
Source: Nat Commun. 2021 May 21;12:3019. doi: 10.1038/s41467-021-23305-7 (PMC8139959; doi:10.1038/s41467-021-23305-7)
Supplement: Supplementary file 1 — Supplementary Information [file 41467_2021_23305_MOESM1_ESM.pdf]

Supplementary Information (SI)

Shape Control of Size-Selected Naked Platinum Nanocrystals

Yu Xia[1,2 ^], Diana Nelli[3^], Riccardo Ferrando[3*], Jun Yuan[4], Z. Y. Li[1*]

[1]School of Physics and Astronomy, University of Birmingham, Edgbaston, Birmingham B15 2TT, U.K.

[2]Department of Materials Science and Engineering, Southern University of Science and Technology, Shenzhen, Guangdong 518055, China

[3]Dipartimento di Fisica and CNR/IMEM, Università degli Studi di Genova, Via Dodecaneso 33, 16146, Genova, Italy

[4]Department of Physics, University of York, Heslington, York, YO10 5DD, U.K.

^ These authors contributed equally to this work.
* Correspondence to: *Z.Li@bham.ac.uk; ferrando@fisica.unige.it or jun.yuan@york.ac.uk*

Supplementary Discussion (SD):

SD-1 Excess Energy of Different Structural Motifs
SD-2 Influence of He Flow Rate on the Formation of Pt Nanoparticles
SD-3 Procedures of Capturing Images for Shape Identification and Their Uncertainty
SD-4 Tetrahedron and Octahedron Image Libraries
SD-5 Examples of Other Structures
SD-6 Molecular Dynamics Simulations for Different Sizes and Temperatures
SD-7 Growth Simulation at 600K and Flux of 0.1 Atoms/ns
SD-8 Growth Simulation at 800 K and Flux of 0.1 Atoms/ns
SD-9 Growth Simulation of the Tetrahedral Tips
SD-10 Simulation of the Tetrahedral Tips

including:

Supplementary Figure 1 Excess energy of nanoparticles with different structural motifs and schematic representation of the growth sequence.
Supplementary Figure 2 Influence of He flow rate on the formation of Pt nanoparticles
Supplementary Figure 3 The procedure of imaging Pt nanoparticles for structure identification.
Supplementary Figure 4 Identification and statistical analysis of shaped nanoparticles
Supplementary Figure 5 Libraries of HAADF-STEM images
Supplementary Figure 6 Representative HAADF-STEM images of experimental Pt nanoparticles with 'other structures'
Supplementary Figure 7 Growth simulation at 600 K and flux of 0.1 atoms/ns
Supplementary Figure 8 Growth simulation at 800 K and flux of 0.1 atoms/ns.

Supplementary Figure 9 Growth of the tetrahedral tips.
Supplementary Figure 10 Snapshots from a growth simulation at T=700 K and deposition rate of 1 atom/ns
Supplementary Figure 11 Snapshots from growth simulations at T=900 K and deposition rate of 1 atom/ns.

and

Supplementary Table 1 Comparison between tetrahedra/truncated tetrahedra and truncated octahedra of the same atomic size (*N*)
Supplementary Table 2 Comparison Sampling Data for Nanoparticle Shape Identification
Supplementary Table 3 Final structures of molecular dynamics simulations at different temperatures

SD-1 Excess Energy of Different Structural Motifs

In Supplementary Figure 1, we report the excess energy for regular truncated octahedra, octahedra, truncated tetrahedra and tetrahedra of sizes up to 5000 atoms. Different degrees of symmetric truncation of the tetrahedron are considered. The results in Supplementary Figure 1 clearly show that regular truncated octahedra are the most energetically stable structures, followed by octahedra, truncated tetrahedra and tetrahedra. Energies are calculated by means of the Gupta potential as specified in the Methods section.

To further confirm the out-of-equilibrium character of the tetrahedral crystalline nanoparticles we compare some perfect complete and truncated tetrahedra to truncated octahedra of the same size (see Supplementary Table 1). The sizes considered are magic sizes for the tetrahedra or truncated tetrahedra but not for the truncated octahedra, which therefore present some defects (rectangular basis, asymmetric truncations, excess atoms accommodated on small islands on the surface). However truncated octahedra are by far more energetically favourable in all cases, as shown by the negative values of the energy difference ΔE and the corresponding different excess energies.

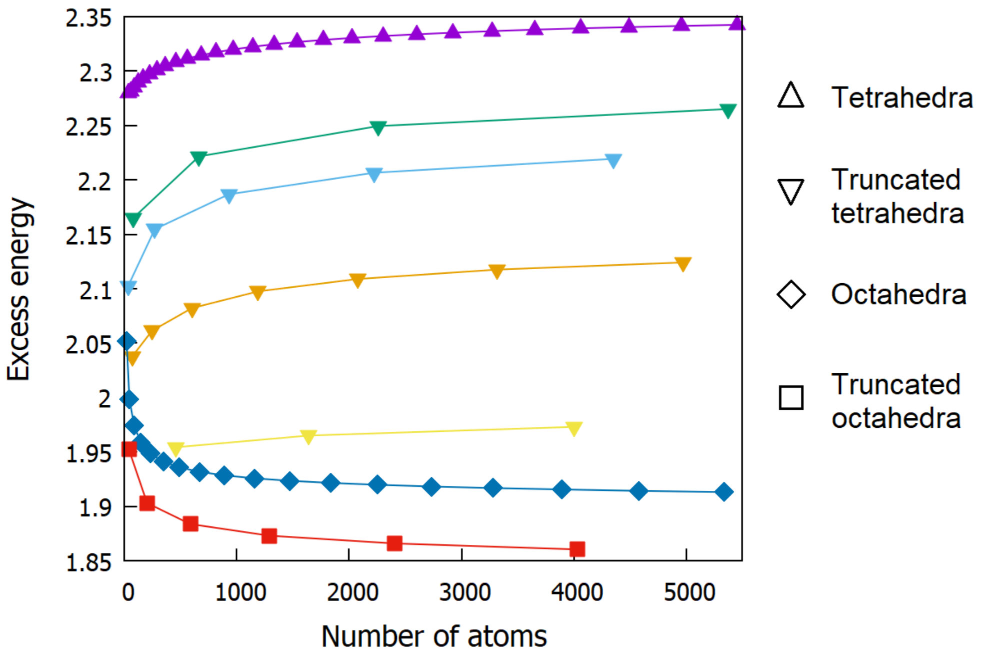

Supplementary Figure 1 | Excess energy of nanoparticles with different structural motifs and schematic representation of the growth sequence. Excess energy E_{exc} is shown as a function of number of atoms (N) of nanoparticles, for regular truncated octahedra (squares), octahedra (diamonds), truncated tetrahedra and tetrahedra (triangles) respectively. Lesser and lesser truncations give tetrahedral structures of higher energy.

N	ΔE	E_{exc}	
		TETRAHEDRON/ TRUNCATED TETRAHEDRON	TRUNCATED OCTAHEDRON
2024	-73.911	2.330	1.869
3654	-112.103	2.338	1.865
5456	-148.935	2.342	1.862
2260	-64.970	2.249	1.872
5376	-124.005	2.265	1.861
2220	-57.223	2.206	1.870
4355	-94.881	2.219	1.864

Supplementary Table 1 | Comparison between tetrahedra/truncated tetrahedra and truncated octahedra of the same atomic size (*N*). We show the energy difference ΔE between truncated octahedra and tetrahedra/truncated tetrahedra and the excess energy E_{exc} of the two structures under comparison, for a range of atomic sizes. We consider three complete perfect tetrahedra (first three rows of the table) and two pairs of truncated tetrahedra with different degrees of truncation (taken from the green and cyan curves of the graph in Supplementary Figure 1, respectively). ΔE and E_{exc} are in eV.

SD-2 Influence of He Flow Rate on the Formation of Pt Nanoparticles

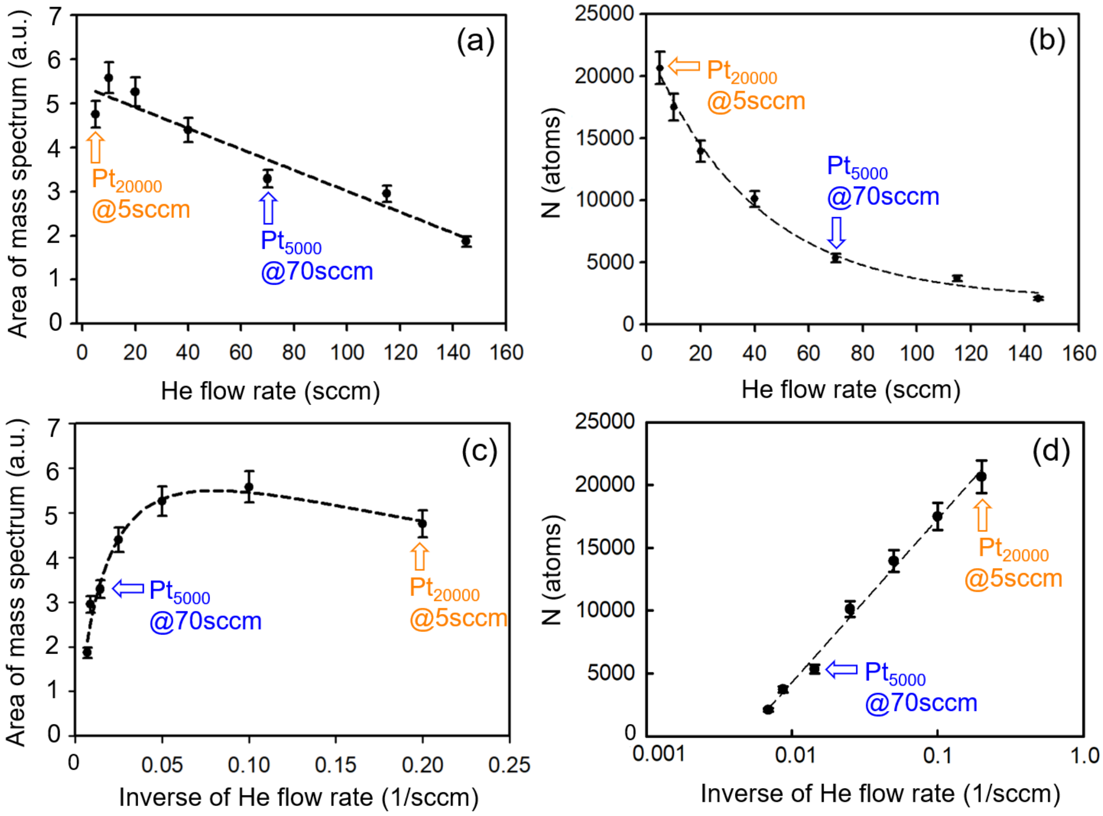

Supplementary Figure 2 | Influence of He flow rate on the formation of Pt nanoparticles shown in Fig. 2b. (a) The area (A) covered by the main peak in mass spectra fitted by Gaussian function as a function of He flow rate showing a linear relationship; (b) The number of atoms at the peak position (*N*) as a function of He flow rate fitted with an exponential function; and (c) The same data as in (a) yet are plotted as a function of inverse of He flow rate, showing a narrow region of nucleation for fast He flow rate and a broad range of no additional nucleation for slow He flow rates. (d) The same data in (b) replotted as a function of the inverse of the He flow rate in the log scale, demonstrating a single relationship. The error bar in the y-axis is from the mass resolution of the mass filter. The blue and orange arrows point to the contrasting growth conditions under which cluster beams showing significant shape transformation from octahedra to tetrahedra. The results demonstrate not only that the shape can be tuned but also the transformation occurs for the nanoparticle beams of similar current but increasing atomic size, suggesting an adatom condensation mechanism.

SD-3 Procedures of Capturing Images for Shape Identification and Their Uncertainty

For shape identification, one small region on the grid is selected randomly and imaged by STEM at low magnification of 600k, for example, as shown in Supplementary Figure 3 (a1) and (b1) for Pt_{5000} and Pt_{20000}. The area is then imaged in sequence with higher magnifications of 2M to include every Pt particle within the area for visual identification, as shown in Supplementary Figure 3(a2), 3(a3) and 3(b2). For those particles cannot be recognised easily by eyes, images with atomic-scale details are taken at even higher magnifications, e.g. 10M in Supplementary Figure 3(b3) and analysed as described in the main text. About 100 Pt nanoparticles are imaged at 10M for Pt_{5000} and Pt_{20000}, respectively. All imaged nanoparticles are classified as three groups: tetrahedra, octahedra, and other structures, and their populations are shown in Fig. 2e.

For assessing the uncertainty of the structure grouping, three regions (R1-R3) in different areas are chosen to repeat the above procedures. Supplementary Table 2 displays the number of Pt nanoparticles with different shapes identified in each area. The variation in the percentage counts of the same shape in the different regions is used to estimate the counting uncertainty.

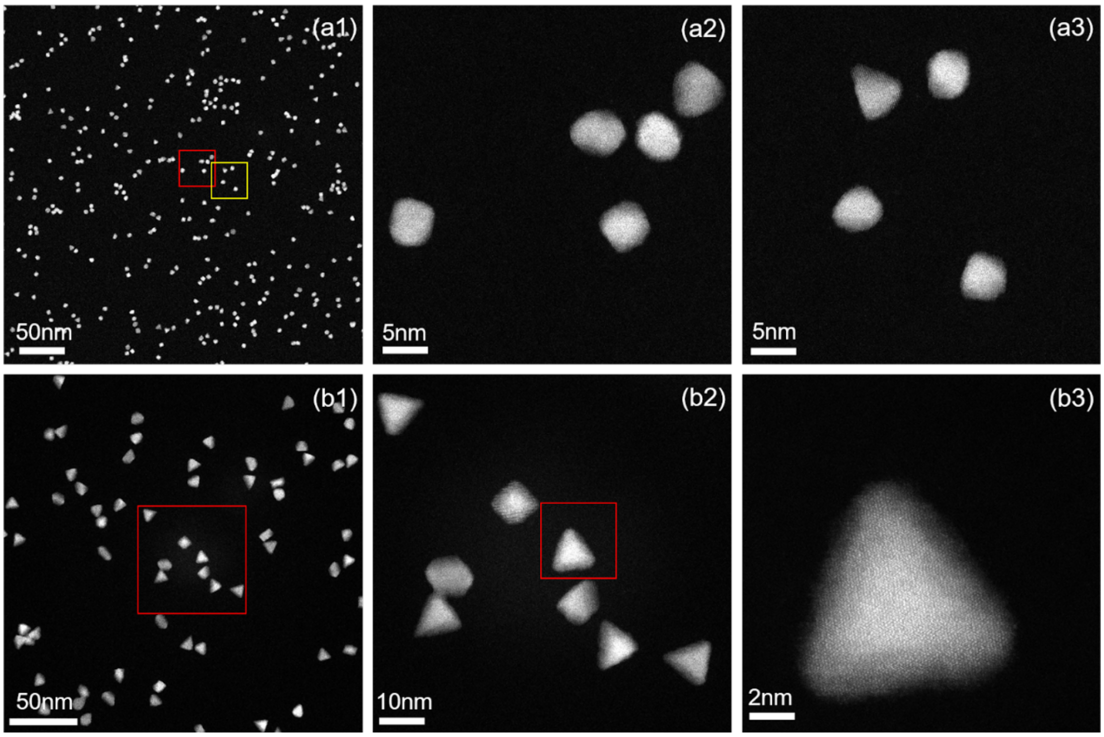

Supplementary Figure 3 | The procedure of imaging Pt nanoparticles for structure identification. (**a1-a3**) Pt_{5000}, and (**b1-b3**) Pt_{20000}.

Supplementary Table 2 Sampling Data for Nanoparticle Shape Identification.

Sample	Regions on the grid (No. of nanoparticles)	Tetrahedron	Octahedron	Others
Pt_{3900} @ 115 sccm	R1 (109)	24%	64%	12%
	R2 (150)	17%	74%	9%
	R3 (100)	19%	75%	6%
	Average (Total no. 359)	20 ±3%	69 ±5%	9 ±3%
Pt_{5000} @ 70 sccm	R1 (284)	19%	73%	8%
	R2 (153)	16%	76%	8%
	R3 (179)	19%	69%	12%
	Average (Total no. 616)	18% ± 2%	73% ± 4%	10% ± 2%
Pt_{10000} @ 40 sccm	R1 (91)	30%	53%	17%
	R2 (125）	29%	57%	14%
	R3 (115)	32%	55%	13%
	Average (Total no.331)	30 ±1%	55 ±2%	15 ±2%
Pt_{20000} @ 5 sccm	R1 (174)	55%	32%	14%
	R2 (329)	50%	40%	10%
	R3 (250)	51%	39%	10%
	Average (Total no. 753)	52% ± 3%	36% ± 4%	12% ± 2%

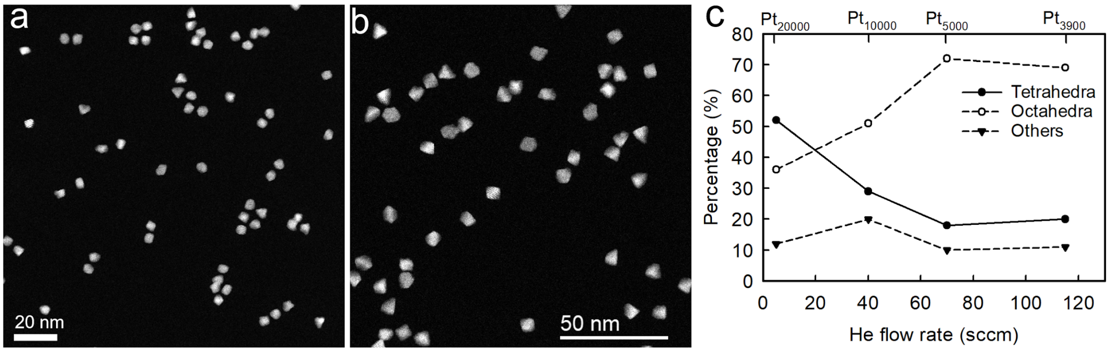

Supplementary Figure 4 | Identification and statistical analysis of shaped nanoparticles. (**a**) Pt_{3900} nanocrystals; (**b**) Pt_{10000} nanocrystals; (**c**) The percentages of Pt nanocrystals with tetrahedral, octahedral, or others' shapes, prepared with different He gas flow rates. The sputtering power was 115 W and Ar gas was 90 sccm.

SD-4 Tetrahedron and Octahedron Image Libraries

The deposited Pt nanoparticles with the same size and shape can display different projected images. To help with identification of the experimental HAADF-STEM images, we generate structural libraries for tetrahedron and octahedron, showing simulated STEM images of the same models but rotated systematically along the *x*- and *y*-axis, as shown in Supplementary Figure 5.

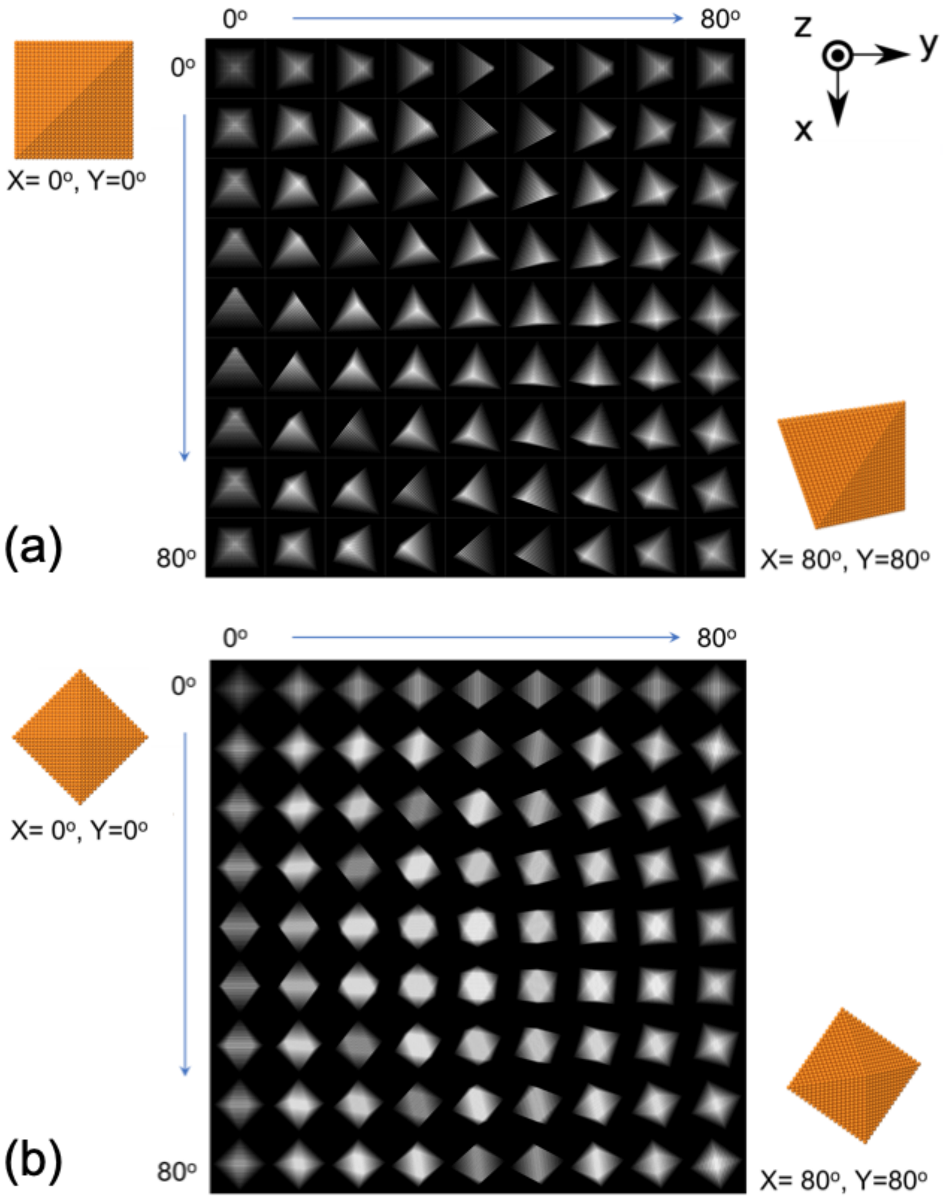

Supplementary Figure 5 | Libraries of HAADF-STEM images for atomic models of (**a**) tetrahedral nanoparticles with 4459 atoms and (**b**) decahedral nanoparticle containing 4579 atoms respectively. Both 4459 and 4579 are full shell atom numbers (magical numbers) for tetrahedron and octahedron respectively. STEM images of rotating the atomic models along *x*-axis and/or *y*-axis with 10° are shown.

SD-5 Examples of Other Structures

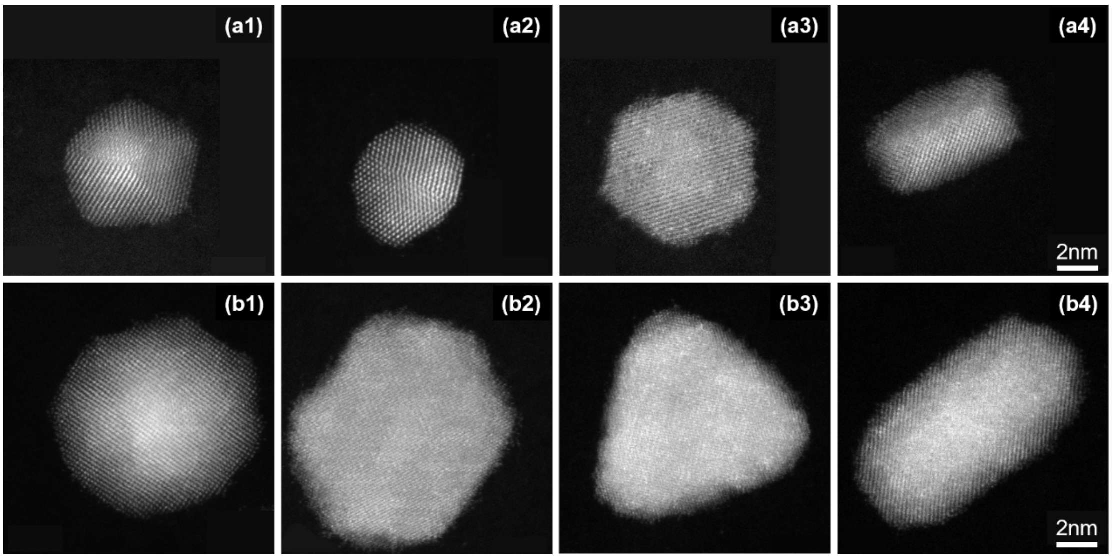

Supplementary Figure 6 | Representative HAADF-STEM images of experimental Pt nanoparticles with 'other structures' as described Fig.2. The images at the upper row are Pt_{5000} nanoparticles and at the bottom row are Pt_{20000} nanoparticles. (a1) a regular five-fold decahedron; (a2) a particle with a twin structure; (a3), (b2) and (b3) particles with flat hexagonal shape and triangular shape; (a4) and (b4) elongated particles; (b1) a rounded shape particle with fcc structure. These "other structures or shapes" occupy low populations, compared with octahedra and tetrahedra. Few structures with elongated or hexagonal shape are also grown in the molecular-dynamics simulations.

SD-6 Molecular Dynamics Simulations for Different Sizes and Temperatures

The final structures observed in the growth MD simulations are reported in Supplementary Table 3. Sequences from representative simulations at temperatures of 600 and 800 K are given in Supplementary Figure 7 and Supplementary Figure 8, respectively. The growth rates of the tetrahedral tips of Supplementary Figure 7 are then analyzed in Supplementary Figure 9. Sequences of the growth of larger clusters are shown in Supplementary Figure 10 and Supplementary Figure 11. In the latter case, the simulations are made by the version of our code running on graphic processor units (GPUs).

In Supplementary Table 3 we consider seven different growth temperatures (300, 400, 500, 600, 700, 800 and 900 K) and, for each temperature, two deposition rates (1 atom/ns and 0.1 atom/ns) are considered. For all pairs (temperature, deposition rate), five independent simulations are made, for a total of 70 independent simulations.

From the results in Supplementary Table 3, it turns out that, depending on simulation temperature and deposition rate, different final outcomes of the growth process are observed. At the lowest temperature, irregular structures with rough surface are observed at the end of the simulations. In the temperature range 400-600 K, tetrahedra become the most frequently observed motif, with more and more regular surfaces as temperature increases. From 700 K onwards, growth takes place mostly within the octahedral motif, with truncations becoming more and more present at the highest temperatures. A few twin structures are also grown, in agreement with the experimental observations. At 600 K, a hexagonal structure, of the type shown in the experimental images of Supplementary Figure 6(a3) and 6(b2), is grown. The only octahedral structure grown at 500 K is indeed quite elongated, with a shape resembling that of Supplementary Figure 6(a4) and 6(b4).

The effect of slowing down the deposition rate is to produce structures that are somewhat closer to the equilibrium. This effect is rather clear at low temperatures, while at high temperatures the results seem independent of the growth rate (at least in this interval of rates).

We also made simulations for larger sizes, beginning with a truncated octahedron of 586 atoms and depositing on top of it at a rate of 1 atom/ns to reach size 3000. We made 5 independent simulations at 600 K and at 700 K. At 600 K, we obtained (as final results at 3000 atoms) 4 tetrahedra and 1 hexagonal structure, whereas at 700 K we obtained 4 tetrahedra and 1 octahedron. Snapshots from a sequence at 700 K are given in Supplementary Figure 10.

In Supplementary Figure 11 we show snapshots of simulations of the growth at 900 K. In Supplementary Figure 11a we show the sequence truncated octahedron → octahedron → tetrahedron starting from about 1300 atoms and ending to about 10000 atoms. In Supplementary Figure 11b we show a sequence starting with a perfect octahedron of about 4600 atoms, then evolving to a deeply truncated tetrahedron for sizes around 10000 atoms and finally growing to a sharper tetrahedron close to 15000 atoms. We note that these sequences perfectly match the experimental sizes.

In summary, these results show that, as expected, temperature increase and slowing down of deposition rate decrease the importance of kinetic trapping phenomena at a

given size, thus allowing the production of growth shapes that are closer and closer to equilibrium. On the other hand, temperature increases and slower deposition rates shift the out-of-equilibrium phenomena leading to the growth of tetrahedra to larger sizes.

Supplementary Table 3: **Final structures of molecular dynamics simulations at different temperatures** from 300 to 900 K and for two deposition rates (1 and 0.1 atom/ns). Simulations start from a truncated octahedral structure of 201 atoms and are stopped when the nanoparticle size reaches 1001 atoms. As regards twins and hexagons, the only structure with hexagonal shape (with a shape very similar to that of the experimental structure of Supplementary Figure 6(a3) and 6(b2) is the one grown at 600 K, whereas the structures grown at 800 and 900 K are triangular twins.

Temperature and deposition rate	Octahedral motif	Tetrahedral motif	Twins and hexagons	Disordered
300 K, 1 atom/ns	0	0	0	5
300 K, 0.1 atom/ns	0	1	0	4
400 K, 1 atom/ns	0	1	0	4
400 K, 0.1 atom/ns	0	5	0	0
500 K, 1 atom/ns	0	5	0	0
500 K, 0.1 atom/ns	1	4	0	0
600 K, 1 atom/ns	2	2	1	0
600 K, 0.1 atom/ns	1	4	0	0
700 K, 1 atom/ns	4	1	0	0
700 K, 0.1 atom/ns	5	0	0	0
800 K, 1 atom/ns	4	0	1	0
800 K, 0.1 atom/ns	4	0	1	0
900 K, 1 atom/ns	4	0	1	0
900 K, 0.1 atom/ns	4	0	1	0

SD-7 Growth Simulation at 600K and Flux of 0.1 Atoms/ns

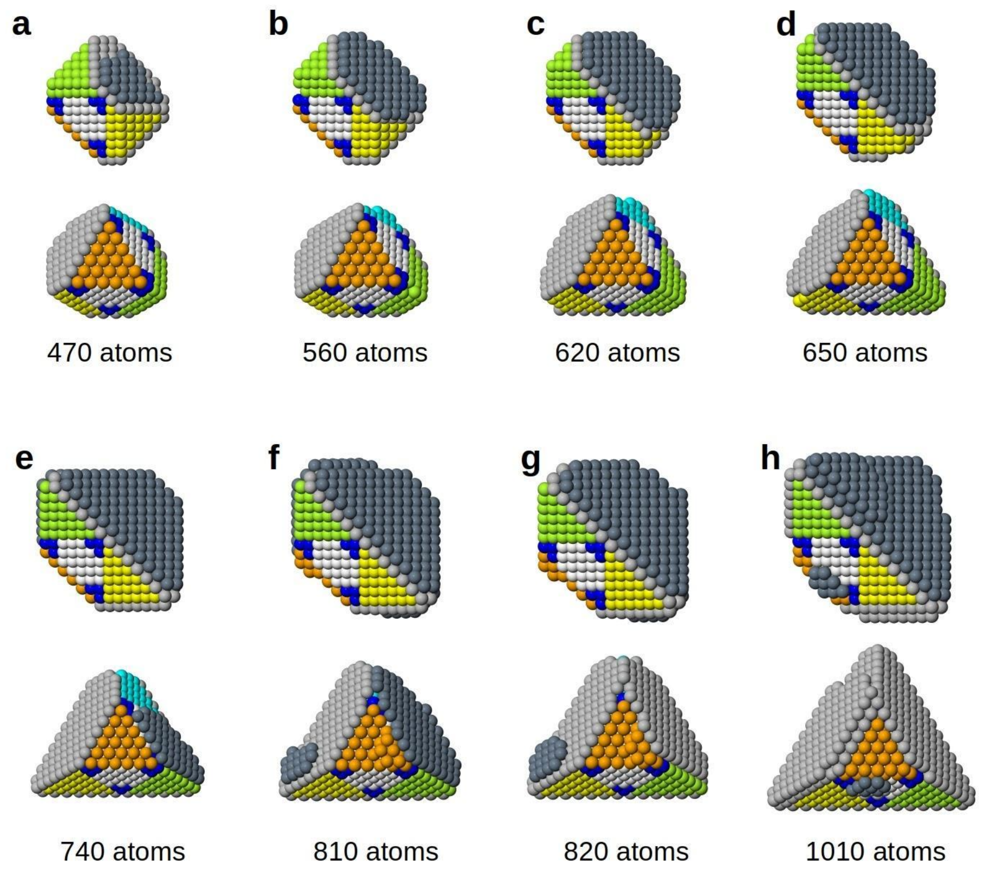

Supplementary Figure 7 | Growth simulation at 600 K and flux of 0.1 atoms/ns. (a-g) Snapshots are taken at eight different intermediate sizes. For each size, the same structure is shown from two different perspectives. Atoms colored in white correspond to the starting truncated octahedron of 201 atoms, while blue atoms are those completing the octahedral tips on top of it, to form an octahedron of 231 atoms. Atoms in yellow, green, orange and cyan correspond to the tetrahedral tips which would grow on that octahedron of 231 atoms, as in the case of the sequence at 400 K of Fig. 3 of the main text. Light grey atoms correspond to further layers in fcc stacking, whereas dark grey atoms are those of islands in hcp stacking. At variance of the sequence at 400 K, growth is still within the octahedral motif up to about a size of (a) 470 atoms, where an island in hcp stacking is formed (atoms in dark grey). (b-d) This island triggers the fast growth of the three tetrahedral tips over four (those in yellow, green and cyan) that have borders in common with it, while the orange tip, which has no faulted islands at its border, does not grow. (e-f) A second faulted island is formed at the border of the orange tip, at its right, and the orange tip starts growing one layer. (g-h) This second faulted island reverts back to fcc stacking, and the growth of the orange tip stops again, no further layers nucleating beyond the second. The final structure is of tetrahedral type, but with three sharp tips and one truncated tip. Note that another small faulted island on the left side of the lower image in (f-g), reverts back to fcc stacking (h) producing a tetrahedron with one more fcc plane.

SD-8 Growth Simulation at 800 K and Flux of 0.1 Atoms/ns

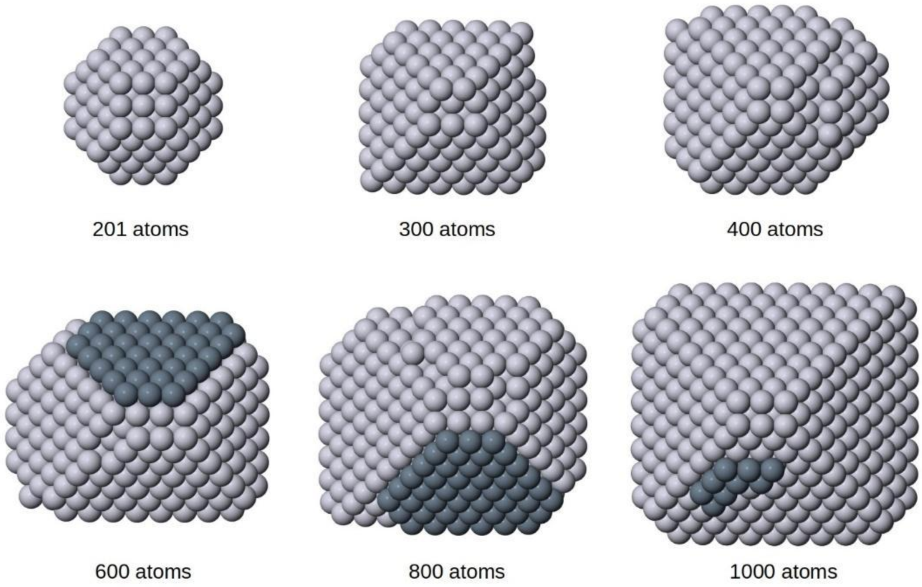

Supplementary Figure 8 | Growth simulation at 800 K and flux of 0.1 atoms/ns. The growth starts from the truncated octahedron of 201 atoms and continues within the octahedral motif up to the final structure at 1000 atoms. All images are taken from the same perspective. Islands in hcp stacking often form (shown by the atoms in dark grey) but they revert back to the fcc stacking as growth continues. At variance with the lower temperature cases, the fourfold adsorption sites formed at the border of faulted islands are not as effective as atom traps, so that tetrahedral tip growth is not started.

SD-9. Growth Simulation of the Tetrahedral Tips

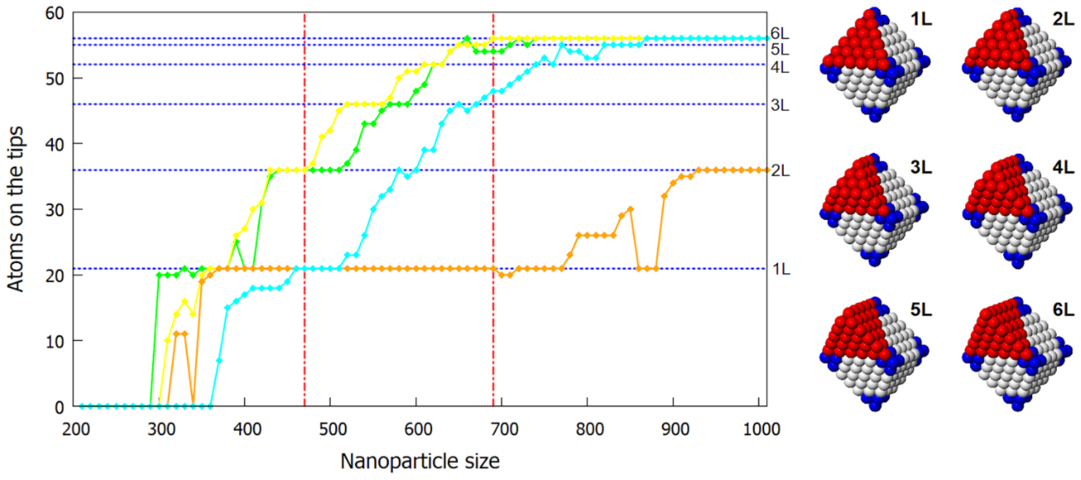

Supplementary Figure 9 | Growth of the tetrahedral tips. The graph refers to the growth sequence of Supplementary Figure 7. The number of atoms on the four tetrahedral tips during the growth is shown, each curve referring to the correspondingly colored tip of Supplementary Figure 6. Tetrahedral tips are made of smaller and smaller triangular layers stacked one on another (see the right part of the figure). Blue horizontal lines on the graph point out the number of atoms at which the different layers of the tips are completed, whereas red vertical lines mark the steps of the growth at which islands in hcp stacking are formed. Before the formation of the first faulted island, the green and yellow tips have completed two layers whereas the cyan and orange tips have completed only one layer. After the formation of the island, the green, yellow and cyan tips grow quickly until they complete the last layer. The growth of the orange tip, which is the only one not connected to the first faulted island, is much slower: the tip is blocked at the first layer until a second faulted island is formed at its border.

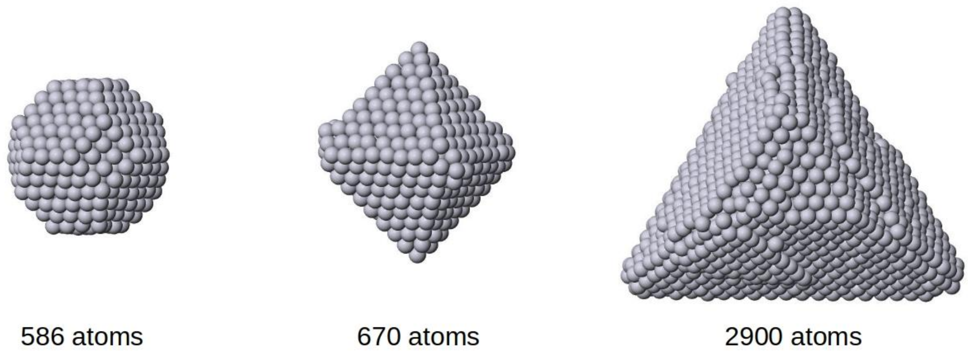

Supplementary Figure 10 | Snapshots from a growth simulation at T=700 K and deposition rate of 1 atom/ns. The simulation is started from a truncated octahedron of 586 atoms, which grows to an octahedron and then to a tetrahedron of 2900 atoms.

SD-10. Growth Simulation of the Tetrahedral Tips

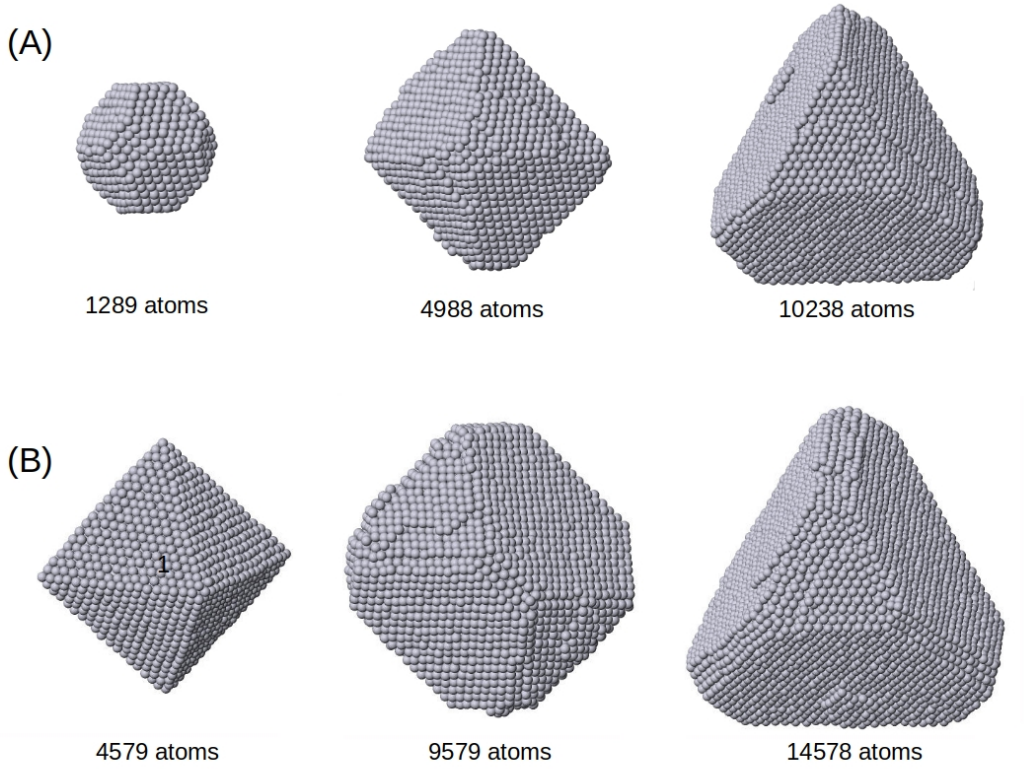

Supplementary Figure 11 | Snapshots from growth simulations at T=900 K and deposition rate of 1 atom/ns. (A) the simulation is started from a truncated octahedron of 1289 atoms, which grows to a somewhat asymmetric octahedron for sizes around 5000 atoms and then evolves towards a tetrahedral shape at about 10000 atoms. (B) The simulation starts from a perfect tetrahedron of about 4600 atoms, then it evolves to a deeply truncated tetrahedron at about 10000 atoms, which becomes sharper growing up to sizes close to 15000 atoms.
